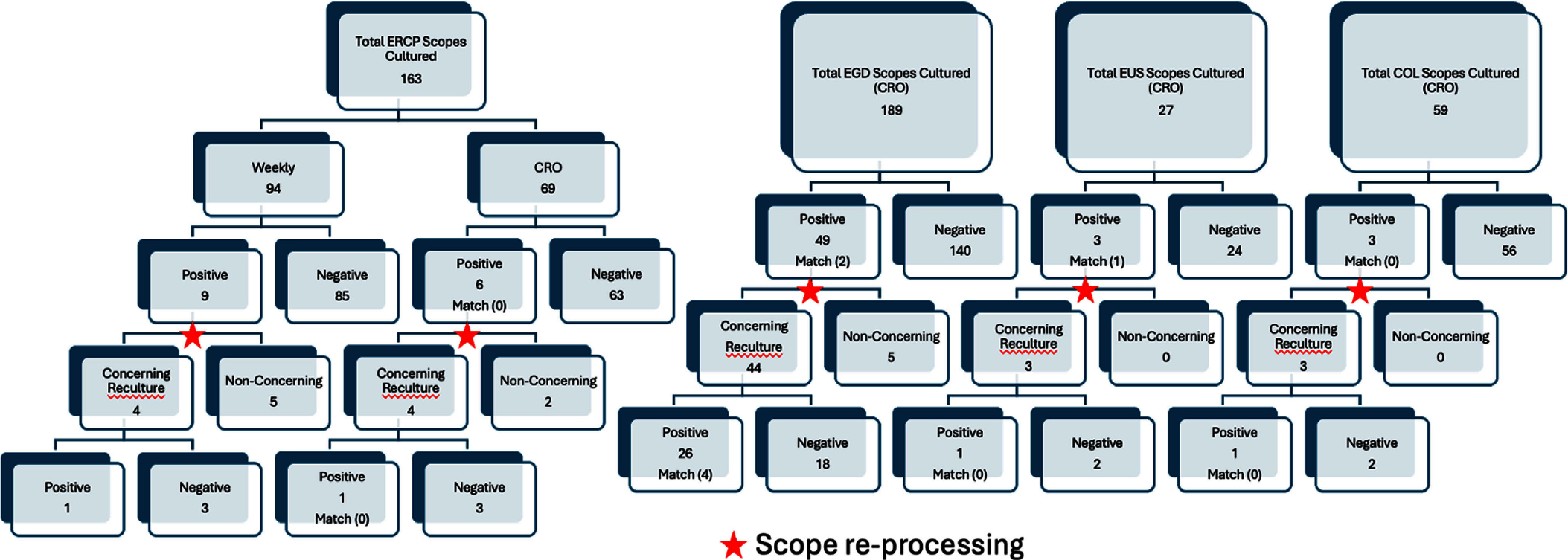# Endoscopic Weekly and Carbapenem-Resistant Organisms Surveillance Program Implementation and Assessment between 2019 and 2024

**DOI:** 10.1017/ash.2025.327

**Published:** 2025-09-24

**Authors:** Court Desmond, Faith Fursman, Kimberly Blanton, Kevin Hatton, Rachel Howard, Olafsson David, Sean McTigue, Nicholas Van Sickels, Sherese Hinton, Takaaki Kobayashi

**Affiliations:** 1 University of Kentucky; 2 University of Kentucky Healthcare; 3 UK Healthcare; 4 Employee; Deborah Flomenhoft

## Abstract

**Background:** The risk of bacterial transmission through gastrointestinal endoscopes remains a critical concern in healthcare-associated infections, driven by the complex design of endoscopes and potential lapses in reprocessing protocols. Contaminated endoscopes can serve as vectors for multidrug-resistant organisms, posing significant threats to patient safety. Current U.S. guidelines for endoscope reprocessing and infection control do not mandate routine surveillance sampling; however, select facilities have successfully adopted routine surveillance cultures to monitor reprocessing efficacy. The Centers for Disease Control and Prevention (CDC) has published protocols to support facilities choosing to implement such practices, emphasizing the importance of identifying persistent transmission risks. **Methods:** This retrospective study was conducted at the University of Kentucky Healthcare (UKHC), a 1,086-bed academic medical center, from January 1, 2019, to June 30, 2024. UKHC implemented a surveillance program in July 2016 targeting endoscopic retrograde cholangiopancreatography (ERCP), esophagogastroduodenoscopy (EGD), endoscopic ultrasound (EUS), and colonoscopy endoscopes. Weekly cultures were performed on ERCP scopes, while targeted cultures were conducted on all four scope types used for patients colonized with carbapenem-resistant organisms (CRO). Following manufacturer instructions for use (IFU), post-reprocessing cultures were performed, and pathogens were categorized as concerning or non-concerning based on CDC protocols. Manual chart reviews identified CRO-colonized patients, and match rates were calculated by comparing endoscope culture results with patient isolates. **Results:** A total of 163 ERCP scopes were cultured, comprising 94 from weekly surveillance and 69 from CRO-targeted surveillance (Figure 1). Weekly surveillance yielded a 9.6% (9/94) positivity rate, while CRO-targeted surveillance showed an 8.7% (6/69) positivity rate. Among six positive samples, no matching CRO was identified. Among 189 EGD scopes subjected to CRO-targeted surveillance, the positivity rate was 25.9% (49/189), with a 4.1% (2/49) match rate to patient isolates. For 27 EUS scopes, the positivity rate was 11.1% (3/27), with a 33.3% (1/3) match rate. Among 59 colonoscopy scopes, the positivity rate was 5.1% (3/59), with no matches to patient isolates. **Conclusions:** The UKHC surveillance program highlights ongoing risks of bacterial transmission despite adherence to manufacturer-recommended reprocessing protocols. Scopes yielding concerning organisms underwent additional reprocessing to mitigate patient risk. All scopes with positive organisms after the second reprocessing were sent back to the manufacturer. Surveillance programs provide valuable insights into disinfection efficacy, helping to identify gaps and guide infection prevention strategies. Further refinement and standardization of surveillance protocols are essential to mitigate transmission risks associated with gastrointestinal endoscopy and improve patient safety on a broader scale.

**Figure:**